# Nutrients Mediate Intestinal Bacteria–Mucosal Immune Crosstalk

**DOI:** 10.3389/fimmu.2018.00005

**Published:** 2018-01-24

**Authors:** Ning Ma, Pingting Guo, Jie Zhang, Ting He, Sung Woo Kim, Guolong Zhang, Xi Ma

**Affiliations:** ^1^State Key Laboratory of Animal Nutrition, Department of Animal Nutrition and Feed Science, China Agricultural University, Beijing, China; ^2^Animal Husbandry and Veterinary Department, Beijing Vocational College of Agriculture, Beijing, China; ^3^Department of Animal Science, North Carolina State University, Raleigh, NC, United States; ^4^Department of Animal Science, Oklahoma State University, Stillwater, OK, United States; ^5^Department of Internal Medicine, University of Texas Southwestern Medical Center, Dallas, TX, United States; ^6^Department of Biochemistry, University of Texas Southwestern Medical Center, Dallas, TX, United States

**Keywords:** nutrients, bacteria, mucosal immunity, intestine, crosstalk

## Abstract

The intestine is the shared site of nutrient digestion, microbiota colonization and immune cell location and this geographic proximity contributes to a large extent to their interaction. The onset and development of a great many diseases, such as inflammatory bowel disease and metabolic syndrome, will be caused due to the imbalance of body immune. As competent assistants, the intestinal bacteria are also critical in disease prevention and control. Moreover, the gut commensal bacteria are essential for development and normal operation of immune system and the pathogens are also closely bound up with physiological disorders and diseases mediated by immune imbalance. Understanding how our diet and nutrient affect bacterial composition and dynamic function, and the innate and adaptive status of our immune system, represents not only a research need but also an opportunity or challenge to improve health. Herein, this review focuses on the recent discoveries about intestinal bacteria–immune crosstalk and nutritional regulation on their interplay, with an aim to provide novel insights that can aid in understanding their interactions.

## Introduction

Intestine plays an indispensable role in the origin of diseases as the shared principal junction of nutrients, microbiota, and immune response. There is a common view that commensal bacteria that count may more than 100 trillion in our gastrointestinal tract (1), influence the host health *via* influencing the operation of immune system, and they are essential for the development and normal operation of immune system. A delicate homeostasis between the commensal bacteria and the host immunity is also closely bounded up with physiological disorders and diseases mediated by the break of homeostasis, such as inflammatory bowel disease (IBD), metabolic syndrome, diabetes, allergy, and cancer (2). Modifying the bacterial community is becoming a potent way for diseases prevention and treatment. Furthermore, it is not to be ignored that bacterial species can produce costly and sufficient extracellular metabolites to benefit and affect host health only when a sufficient biomass of bacteria can benefit from the public goods of host, such as nutrients (3).

Hence, it is potential to modify immune response to attenuate and treat the aforementioned diseases *via* modulating gut bacterial composition and functions. Nutrition intervention, probiotics supplementation and bacterial transplantation are three alternative protocols to alter gut bacterial community nowadays, of which, nutrition intervention is a preferential choice in view of its high accessibility and security (4). Thus, to understand the cooperation and communication of bacteria and host immunity, nutrients should be the first factor need to be thought about.

Considering that the intestine is the shared site of nutrient digestion, microbe habitat, and immune cell location, this geographic proximity contributes to a large extent to their interaction. It is necessary for us to focus on the recent discoveries about intestinal bacteria–immune crosstalk and nutritional regulation on their interplay with an aim to provide novel insight that can aid in understanding of their interaction.

## The Crosstalk Between Intestinal Bacteria and Mucosal Immune

The immune system consists of two parts: innate and adaptive immune system. They work cooperatively to defend the body against pathogen invasion and immune disorders. Generally, the immune response is produced throughout the whole body. The interaction and shared signal pathways exist between mucosal immune and intestinal bacteria. Immune signaling plays an essential role in managing the microbiota to maintain health homeostasis in the gut. Correspondingly, the mucosal immune also responses to the alteration of microbiota and the stimulation of microbial metabolites. Here, we focus on the intestine to understand the bidirectional mediation of gut bacteria and intestinal innate and adaptive immune responses.

### Interaction between Gut Bacteria and Innate Immune System

Physical barriers, innate immune cells and molecules constitute the innate immune system, which is the first line to defend against pathogen infection. Within the intestine, these bacteria are controlled by the activity of neutrophils and macrophages *via* pattern recognition receptors (PRRs) to recognize and respond to abnormal changes of the microbial landscape. PRRs identify microorganism-associated molecular patterns (MAMP), mainly including peptidoglycan, flagellin, lipopolysaccharide (LPS), and nucleic acid structures of microbes (5). Some bacterial metabolites from nutrient fermentation are also recognized by PRRs, such as butyrate.

**Figure 1:**
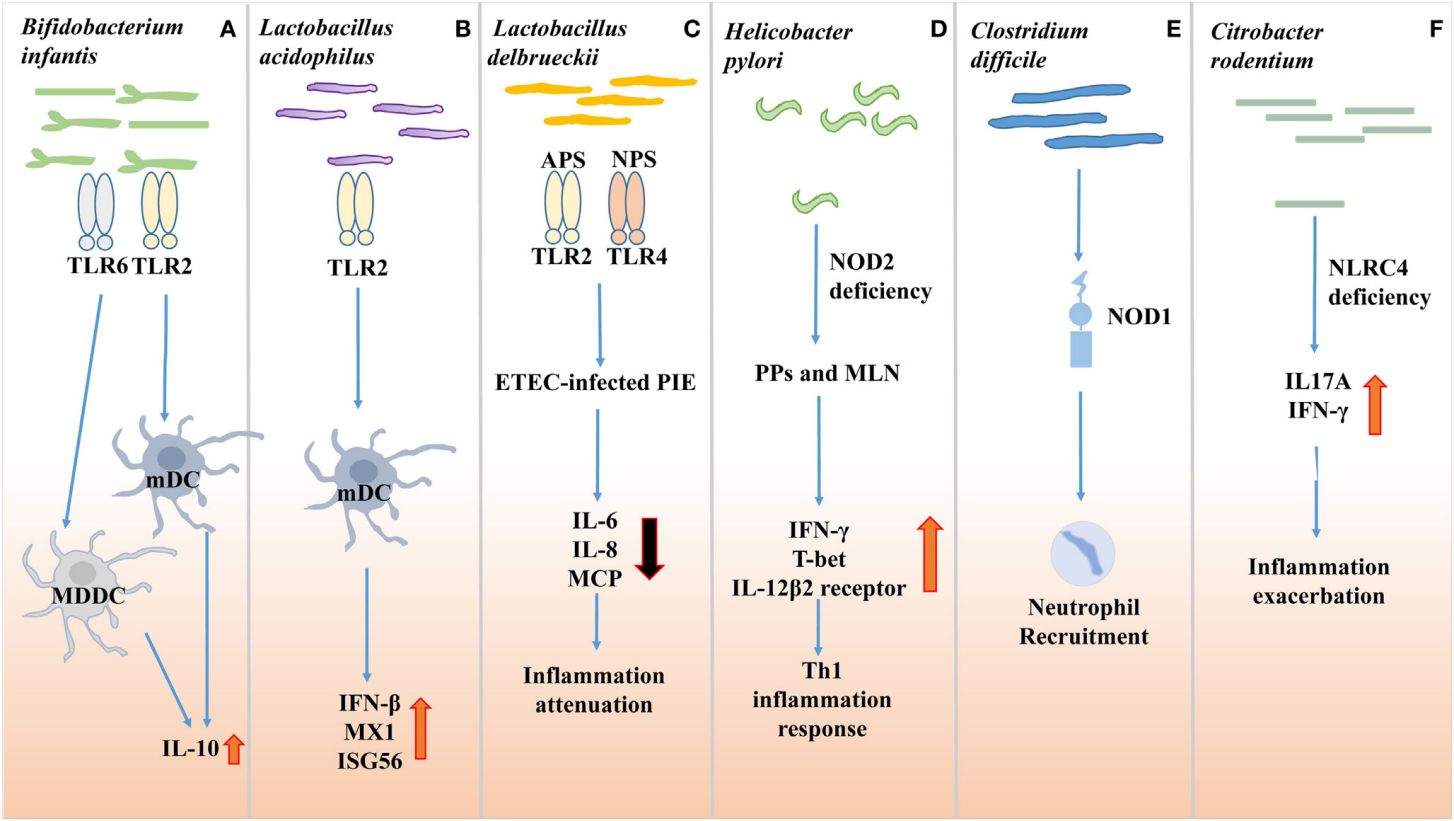
Effects of gut microbes on innate immune receptor. **(A)** *Bifidobacterium infantis* 35624 treatment increases IL-10 secretion through the TLR2/TLR6 pathway in human myloid dendritic cell (mDC) and monocyte-derived DC (MDDC), while IL-10 secretion in plasmacytoid DC (pDC) is TLR9 dependent. **(B)** *Lactobacillus acidophilus* NCFM facilitates murine myeloid DC to express antiviral genes, such as myxovirus resistance 1, interferon (IFN)-β and interferon-stimulated gene 56 (Isg56), *via* TLR2 pathway. **(C)** *Lactobacillus delbrueckii* subsp. *delbrueckii* TUA4408L (Ld) response against Enterotoxigenic *Escherichia coli* (ETEC) 987P infection in porcine intestinal epithelial cells. Acidic extracellular polysaccharide (APS) and neutral extracellular polysaccharide (NPS) of Ld attenuate inflammation dependent on TLR2 and TLR4, respectively. **(D)** Non-invasive *Helicobacter pylori* infection in NOD2−/− mice relies on NOD2 signaling to induce Th1 inflammation response. **(E)** Non-invasive *Clostridium difficile* infection recruits neutrophils to infection sites *via* nucleotide-binding oligomerization domain protein 1 (NOD1). **(F)** *Citrobacter rodentium* induces inflammation exacerbation in NLRC4−/− mice by producing IL17A and IFN-γ.

So far, PRRs in the innate immune system sense microorganisms through conserved molecular structures. PRRs consist of the toll-like receptors (TLRs), the nucleotide-binding oligomerization domain (NOD)-like receptors (NLRs), the retinoic acid inducible gene 1 (RIG-I)-like receptors (RLRs), the C-type lectin receptors (CLR), the absent in melanoma 2-like receptors and the 2’-5’oligoadenylates synthesis (OAS)-like receptors (OLRs) (6). Several cellular compartments contribute to the expression of these sensors, and they together constitute a continuous surveillance system for the presence of microbes in gut. Here, we view PRRs as the indispensable components in innate immune system although some PRRs have been reported to participate in adaptive immune response simultaneously (7), and focus on two vital PRRs subfamilies, TLR and NLR, which are very prominent examples to understand bacteria–host interaction (Figure 1).

#### The Crosstalk between Intestinal Bacteria and Mucosal Barrier

The intestinal mucosa is a dynamic interface containing an epithelial monolayer designed to separate the gut-associated lymphoid tissue from commensal bacteria community (8). Functionally, the intestinal mucosal barrier is the first natural line to defense against pathogen invasion *via* the cooperation of mucus layers, enterocytes, and tight junctions.

##### Pathogens and Mucosal Barrier

Gastric acid and protein hydrolase in the digestive tract destroy most of the pathogens that enter the intestine. However, some stubborn bacteria, such as *Helicobacter pylori* still exist, which survive in the acidic environment by changing the ambient pH. Thus, the effective close connection formed by epithelial cells ought to be a direct barrier to prevent the invasion of pathogens. However, some pathogens, such as many Enterobacteriaceae bacteria, can impair intestinal epithelial integrity and reduce mucus secretion, besides, *Salmonella typhimurium* can cause diarrhea, typhoid fever, and gastroenteritis.

##### The Absence and Existence of Commensal Bacteria Interact with Intestinal Immunity

Furthermore, the bidirectional function between mucosal barrier and intestinal pathogens also requires the interaction with commensal bacteria, which not only contribute to defending against pathogens but also maintaining mucosal integrity and barrier function indispensably. The inner colon mucus layers capacity to separate bacteria from the epithelium is dependent on bacterial colonizers signaling to the host epithelium (9).

Several experiments carried out on germ-free (GF) mice were consistent with the necessity for the existence of commensal bacteria. It was reported that exposing to bacteria was a sufficient way for the intestine to stimulate mucus synthesis (10). Meanwhile, the maturity and abundance of mucus also depended on the intestinal bacteria. Similar researches found that in GF mice, small intestinal mucus was attached to the epithelium and the colonic inner mucus layer was penetrable to bacteria even though the mucus structure was similar to the conventional mice (9, 10). Besides, GF mice were also possessed a lower level of Muc2 O-glycans, which might be correlated with decreased glycosyltransferases responsible for O-glycan elongation (10). Interestingly, the reduction of Muc2 O-glycosylation could in turn hinder bacterial colonization, considering that glycan were utilized by bacteria as attachment sites and energy sources (10).

In addition, in the absence of symbiotic bacteria, the intestinal epithelial cells (IECs) and tight junctions tended to be impaired. The expression of tight junction proteins, the occludin and zonula occludens-1, was observed to reduce in GF mice (10). Compared to the conventional mice, enterocytes on the brush border were more irregularly arranged, and 30% of the enterocytes approximately were possessed of incomplete apical junctional complexes without desmosome in GF mice (11, 12). Fortunately, after colonizing *Lactobacillus*, the arrangement of microvilli was well organized and cytoskeletal microfilaments were anchored in the terminal web (13). The repair function and beneficial effect of probiotics on the intestinal barrier were also confirmed on *Akkermansia muciniphila*, a mucin-degrading bacterium commonly found in human gut. Their potential mechanisms might be associated with regulating the thickness of intestinal mucus in order to maintain the integrity of gut barrier.

#### TLRs Interact with Intestinal Bacteria

Pattern recognition receptors including the TLRs expressed by epithelial cells recognize MAMPs of the commensal bacteria and regulate the crosstalk between intestinal microbes and host (14, 15). Upon MAMP recognition, TLR form a homodimer or heterodimer to recruit the TLR-domain-containing adaptor protein, like myeloid differentiation primary response 88 (MYD88), TIRAP, TRIF, or TRAM, and then activate transcription factors, including nuclear factor kappa-light-chain-enhancer of activated B cells (NF-κB), activator protein 1, interferon-regulatory factor 3 (IRF-3) and IRF-7 (14). The microbiota composition of the host is influenced by the status of TLRs and their adapter proteins (16, 17). Defects in TLR signaling and aberrant immune responses to perturbed endogenous microbiota are a few of the major factors that contribute to the perpetuation of inflammation and tissue injury in patients with IBD (18, 19).

To date, 13 different TLRs have been identified. It has been reported that the expression of ileal TLR4, TLR5, and TLR9 and colonic TLR3, TLR4, TLR6, TLR7, and TLR8 increased in antibiotic-treated mice, while ileal TLR2, TLR3, and TLR6 and colonic TLR2 and TLR9 decreased (17). And TLR2 and TLR4 were upregulated in DSS colitic mice, while the expression of TLR5 decreased and other TLRs remained unchanged (20). The distinctive change of TLR pattern under unhealthy condition may indicate the differentiated functions of TLR members.

##### TLR2

TLR2 is expressed in enteric neurons and smooth muscle cells and senses various components from bacteria, mycoplasma, fungi, and viruses (18). *Lactobacillus acidophilus* NCFM facilitate murine myeloid dendritic cell (mDC) to express antiviral genes, such as myxovirus resistance 1, interferon-β (IFN-β), and interferon-stimulated gene 56, *via* TLR2 pathway (Figure 1B). Another study utilized *Lactobacillus delbrueckii* subsp. *delbrueckii* TUA4408L (Ld) as a mean to limit the response to Enterotoxigenic *Escherichia coli* 987P infection in porcine IECs (Figure 1C). And TLR2 was necessary for Ld to alleviate the inflammatory response. The presence of *Bifidobacterium infantis* 35624 increased IL-10 secretion through the TLR2/TLR6 pathway in human mDC and monocyte-derived DC. A recent study also reported that the polysaccharide A of the *fragile bacillus* could activate TLR2 and promote the secretion of anti inflammatory cytokine IL-10 (21). Besides, TLR2 stimulation can also induce NF-κB activation in inflammatory disease and promote Th17 cell response to enhance the inflammation response (7, 22). So, TLR2 signal can induce both pro- and anti-inflammation responses. And the diverse immune responses depend on its co-receptor and microenvironment (7).

##### TLR4

TLR4 is the first-identified and well-documented TLRs, which is vital in maintaining the fine balance between tolerogenic and inflammatory properties of gut microbiota by regulating innate immunity (23–25). It has been known that TLR4 signal induced by IFN-γ and tumor necrosis factor-α (TNF-α) could promote inflammation development (7). Increased epithelial TLR4 expression is observed in patients with IBD and associated with impaired epithelial barrier and altered epithelial cell differentiation. Studies have confirmed the effective role of TLR4 in microbiota recognition, which recognized LPS on the cell surface with the help of CD14 and MD2 (26, 27) Furthermore, studies also showed that varied expression of TLR4 in different intestinal regions was determined largely by the bacterial composition of that region (23, 28).

In turn, epithelial TLR4 expression also shaped the intestinal microbiota. The overexpression of TLR4 is characterized by bacterial translocation and increased density of mucosa-associated bacteria. More concretely, a decrease in *Fusobacteria* and *Proteobacteria* and an increase in *Firmicutes* in the colonic mucosa are found, which share the similarity with IBD patients (23, 29, 30). In addition, the increase of *Lachnospiraceae* and Gram-positive *Coriobacteria* is also significant. Meanwhile, the reduced number of Paneth cell could be observed, which play a role in limiting the penetration of commensal and invasion of pathogenic bacteria into the mucosa (23, 31). Several researchers also revealed that increased epithelial TLR4 signaling was associated with the altered expression of antimicrobial peptide (AMP) genes. The increased *Reg3g* and *Lyz2* could not only modulate the composition of mucosal microbiota but also provide a first line to defense against mucosal association by bacteria (23, 32). Together, we imply that the bidirectional modulation between TLR4 and intestinal microbes does exist, and can ultimately alter the host susceptibility toward colitis.

##### TLR5

TLR5 is a transmembrane protein that is highly expressed in the gut mucosa and helps defend against infection (33). TLR5 recognizes flagellin and is responsible for detecting the incursive bacteria and eliciting a series of proinflammation response by enhancing IgA production and Th1 and Th17 development (22). It is the other mean for the innate immune recognition, except for NLRC4 (34). TLR5 detects flagellin not only mediates bacterial locomotion (35) but also defends against flagellated pathogens. Unlike NLRC4, TLR5 can specifically help to manage the commensal microbiota, and effectively eliminate patho-bionts which left can promote diseases (36, 37). As reported, the absence of TLR5 on IECs is sufficient to result in low-grade inflammation, metabolic syndrome, proneness to colitis, and dysregulated microbiota composition (33, 38). Specifically, the loss of the flagellin receptor TLR5 causes a small but not insignificant number of bacteria frequently breaching the mucus barrier to contact or surmount the epithelium, and also results in increased levels of flagellated bacteria (39, 40), which would likely have an increased ability to transit through the mucus layer and would, thus, likely be enriched in such perturbing bacteria. Moreover, in this process, higher level of LPS and fecal bioactive proinflammatory flagellin are generated associated with altered microbiota.

Hence, these results above mean that there are exquisite MAMP recognition mechanisms on the TLR to ensure accurate response to bacteria. Meanwhile, the composition and activity of pathogens and commensal bacteria in gut are also regulated by TLR signaling. Above all, different TLRs cooperating with each other in MAMP recognition interact with intestinal bacteria in their relatively independent way.

#### NLRs Interact with Intestinal Bacteria

NLRs are located in the cytoplasm and have two subfamilies: nuclear oligomerization domain proteins subfamily C (NLRC) and nucleotide-binding oligomerization domain (NOD), leucine rich repeat and pyrin domain containing (NLRP) (31). Members of NLRC subfamily are nucleotide-binding oligomerization domain protein 1 (NOD1), NOD2, NLRC4, NLRX1, NLRC3, and NLRC5. The NLRP subfamily consists of 14 proteins with a pyrin domain.

NOD-like receptors are essential for recognizing bacteria to control the healthy gut microenvironment. Multiple studies have shown that mice lacking NOD1, NOD2, or NLPR6 exhibited alteration in their bacterial composition (24, 25, 30–32).

##### NOD1 and NOD2

Among all the NLRs, NOD1 and NOD2 are the first-identified NLR members and play a vital role in pathogen recognition (31). Conversely, the altered composition and translocation of intestinal bacteria will regulate the signaling of NOD1 and NOD2. In mucosal immune system, NOD1 and NOD2 combine with their ligands and then activate the receptor-interacting protein family 2 and NF-κB pathway.

As reported, NOD1 recognizes the d-glutamyl-meso-diaminopimelic acid of Gram-negative bacteria and NOD2 recognizes the muramyl dipeptide, which is the metabolite of peptidoglycan (41). Non-invasive *Helicobacter pylori* infection also relies on NOD2 signaling (Figure 1D) (32). Several studies showed that NOD1 was essential for defending against the non-invasive *Clostridium difficile* and Spi1-deficient *Salmonella* mutant infection (Figure 1E) (32, 33). In the absence of NOD2, the increased *Bacteroides vulgatus* was observed, which exacerbated the inflammation reaction, along with goblet cell dysfunction and abnormal expression of inflammatory genes (32).

Studies also confirmed the management of commensal bacteria *via* NOD. However, some contradictions still existed. NOD1-deficient mice exhibited abnormal expansion of the *Bacteroides*, Clostridiales, Enterobacteriaceae, and the segmented flamentous bacteria (SFB) (32), However, another study reported that there were no significant differences in the relative abundance of targeted bacterial groups between NOD1-deficient mice and wild-type littermates (42). Recently, the adjuvanticity of cholera toxin (CT) depended on recognition of commensal bacteria *via* NOD2 in CD11c^+^ cells and CT enhanced NOD2 activation *via* cAMP/PKA was also reported (43).

##### NLRC4

NLRC4, another member of NLRC subfamily, is reported to be expressed in epithelial crypts and plays an important role in intestinal health. Early NLRC4 sensing of *Citrobacter rodentium* is necessary for regulating its colonization and alleviating intestinal damage (Figure 1F) (34).

After being infected by *Salmonella*, NAIP belonging to IEC is combined with bacterioprotein ligands, such as flagellin (44), and then activates NLRC4 to form the inflammasome. Then the Caspase-1 or Caspase-8 in the downstream will be further activated. As reported, the activation of Caspase-1 leads to the death of IEC, the release of IL-8 and arachidic acid (45). In addition, Caspase-8 sensitized by downstream signal of NLRC4 also has the capacity to eliminate IEC (45). Paradoxically, in this process, the protective and pathogenic effects of Caspase-8 on *Salmonella* infection might both occur (44).

##### NLRP3

Nucleotide-binding oligomerization domain, leucine rich repeat and pyrin domain containing, such as NLRP3, NLRP6, and NLRP12 are the regulators of innate immunity (46). A recent study reported that NLRP6-deficient mice exhibited increased proportion of *Bacteroidete* and TM7 (40). As to NLRP12, patients with ulcerative colitis are associated with lower expression of NLRP12, which is the gene encoding the negative regulator of innate immune. The deficiency of NLRP12 in mice increases the basal inflammation of colon, reduces microbial density, eliminates protective strains of *Lachnospiraceae*, and promotes the prosperity of strains belonging to *Erysipelotrichaceae* and relating to colitis (47).

NLRP3 is an integral part of the inflammasome (48). Intestinal tract with hyperactive NLRP3 is more likely to maintain homeostasis and has a strong resistance to colitis and colorectal cancer (46). Based on our knowledge that increased expression of miR-223 was observed in intestinal inflammatory tissues of IBD, studies found that in the absence of miR-223, NLRP3 expression increased in colonic and medullary cells. Furthermore, miR-223 in the inflammatory mononuclear cells could directly mediate NLRP3, in order to moderate the activity of the inflammatory corpuscle and inhibit colitis (48).

NLRP3 is also crucial in the interaction with intestinal microbiota. Hyperactive NLRP3 can enhance the secretion of IL-1β instead of IL-18 to promote the reconstitution of flora *via* increasing the local AMP. However, this function is only limited in mononuclear phagocytes of lamina propria. Microbial reconstruction will further contribute to inducing Treg and managing to anti inflammation (46).

Due to the discovery of complex and opposing role of many PRRs in immune response, it is necessary to explore the underlying recognition mechanisms of most PPRs under different microenvironments and how they cooperate with each other to differentiate symbiosis from harmful bacteria to maintain the intestinal homeostasis. In summary, most PPRs deficiencies result in an abnormal makeup of the bacterial community. The healthy innate immune system contributes to the optimization of the gut bacterial composition and the dysbiosis of innate immune system may cause an imbalance within the gut bacteria community potentially leading to diseases.

### Interaction between Gut Bacteria and Adaptive Immune System

In general, the adaptive immune system is initiated several days after pathogen infection and produces cytokines and specific antibodies cooperating with innate immune system to defend against subsequent pathogen invasion. The T cells and B cells are the two main immune cells involved in adaptive immune response. Memory T and B cells are further induced to respond to the secondary immune during the adaptive immune response.

In the intestine, the pathogens and commensal bacteria are both effective stimuli to induce the adaptive immune response. Conversely, the adaptive immune system is also a powerful weapon to resist pathogen invasion and regulates symbiotic flora. An aberrant and irrational state of gut bacteria community can be caused by the damage of the adaptive immune system. In the following section, we focus on the bidirectional effect on altered bacteria and adaptive immune response, including T cells differentiation and secretory immunoglobulin A (SIgA) secretion.

#### Bacteria and T Cells Differentiation

Th1, Th2, Th17, Treg, and cytotoxic lymphocyte cells are the principal effector T cells to regulate the adaptive immune response *via* cytokines production. The Th17 cells produce proinflammatory cytokines, such as IL-17, IL-21, and IL-22, to enhance inflammation. The Treg cells secrete the anti-inflammatory cytokine IL-10 to attenuate inflammation. The dynamic balance between Th17 and Treg cell differentiation is mediated by regulation of cytokine, such as IL-6, IL-21, and IL-2 (49, 50).

**Figure 2:**
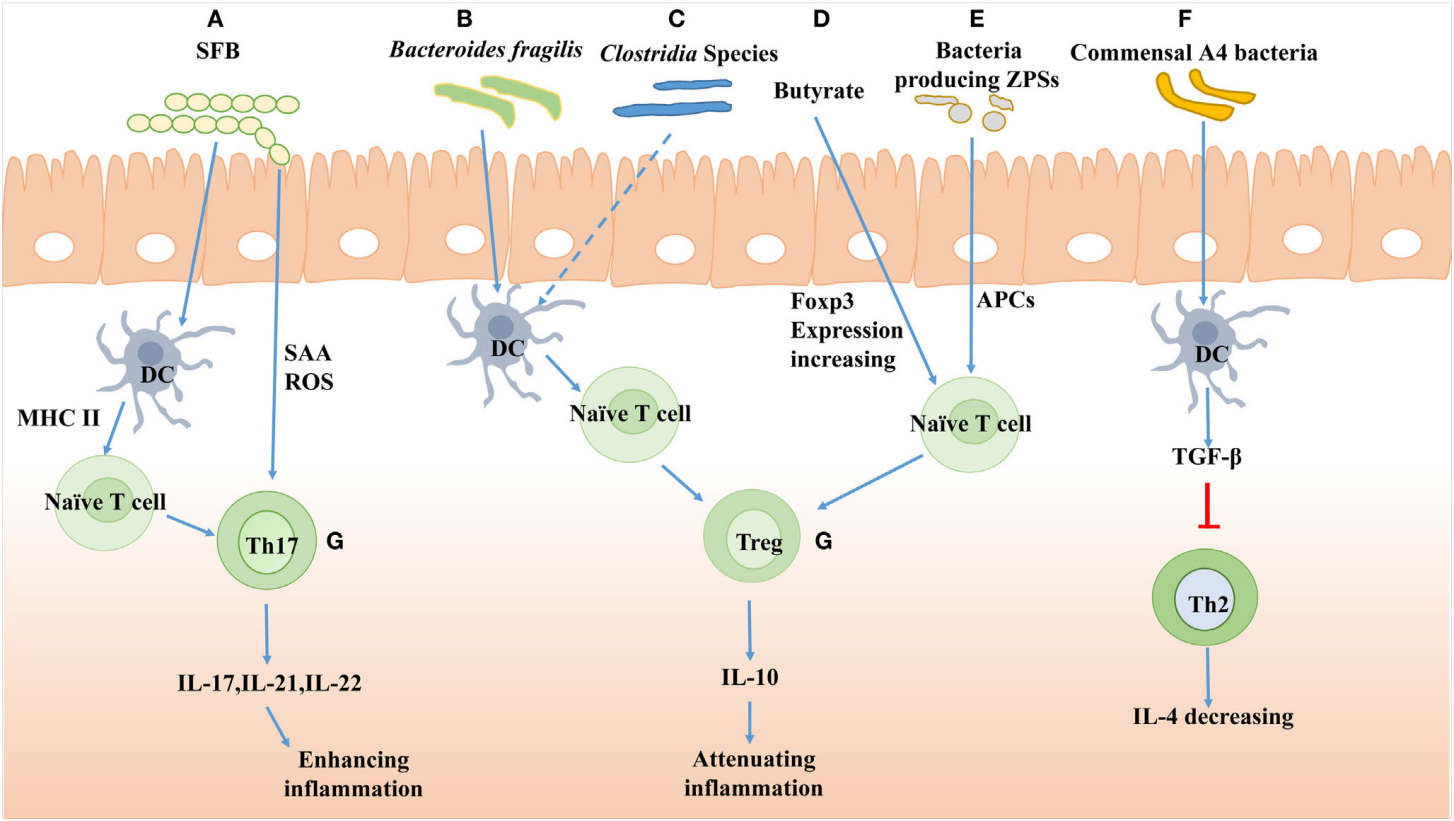
The effects of gut bacteria on T cells differentiation. **(A)** The polysaccharide A of segmented filamentous bacteria (SFB) induces Th17 cells differentiation and the antigen of SFB presented by dendritic cell (DC) is dependent on major histocompatibility complex II (MHCII). SFB adhesion induces Th17 accumulation by producing serum amyloid A (SAA) and reactive oxygen species (ROS). **(B)** The *Bacteroides fragilis* improves Treg cells differentiation mediated by PSA-activated DC. **(C)** *Clostridia* species induces Tregs accumulation presumably by cooperating with DC in the colon. **(D)** Butyrate, a large intestinal bacterial metabolite, drives colonic expansion of Treg cells in mice by reinforcing histone H3 acetylation in the promoter and conserving non-coding sequence regions of the Foxp3 locus. **(E)** Bacteria-producing Zwitterionic capsular polysaccharides (ZPS) can stimulate differentiation of Treg cells and IL-10 production dependent on antigen-presenting cell (APC). **(F)** Commensal A4 bacteria of *Lachnospiraceae* family inhibit Th2 cells production by increasing transforming growth factor-β (TGF-β) production of DC. **(G)** The Th17 cells produce IL-17, IL-21, and IL-22 to enhance inflammation response, while the Treg cells produce IL-10 to attenuate inflammation.

Within the intestine, some bacteria or certain known bacterial mixes can affect the T cell generation and shape its subset. Recent studies showed that SFB primed and induced Th17 cells differentiation locally in the lamina propria. In addition, SFB adhesion to enterocytes induced Th17 accumulation by producing serum amyloid A and reactive oxygen species (51). Furthermore, the antigen of SFB presented by DC was dependent on MHCII (52) (Figure 2). As to some commensal bacteria, a member of the *Lachnospiraceae* family, commensal A4 bacteria, was found to hinder Th2 cells development by inducing the transforming growth factor-β (TGF-β) production through its CBir1 antigen (Figure 2) (53). Several groups also have studied the effect of *Clostridia* colonization on T cell differentiation and reported that the *Clostridia* could induce the expansion of Treg cells to suppress the inflammatory response of colitis mice (Figure 2) (54, 55). On the contrary, in GF mice, the colonized gut bacteria and LPS-rich sterile diet induced T and B cell proliferation and differentiation in PP and MLN, especially the CD4^+^ Foxp3^+^T cells in MLN (56).

Furthermore, the products of bacteria, such as polysaccharide, could also affect T cell differentiation. Polysaccharide A (PSA) from *Bacteroides fragilis* promoted Treg cell secretion and then suppressed Th17 activity to reinforce its intestinal colonization (Figure 2) (57). A genomic screen for bacteria encoding for Zwitterionic capsular polysaccharides (ZPS), the bacterial product which can activate T cells function, was conducted. The lysates of ZPS-producing bacteria could stimulate differentiation of Treg cells and IL-10 production, which depended on antigen-presenting cells (APC) (Figure 2) (58).

Moreover, the deficiency of T cell also exhibits altered bacteria (59). As reported, Disheveled 1 (Dvl-1) is an important protein of the Wnt/β-catenin pathway (60), which controls the proliferation of T cell progenitors and regulates T cell development and Treg cell activation (61–63). In *Dvl-1* knockout mice, the gut bacterial composition is altered through the promotion of opportunistic pathogen growth, such as *Helicobacter mastomyrinus*, and hinderance of commensal bacteria growth (64).

Based on above researches, it can be speculated that regulation of T cell differentiation is a mechanism for gut commensal bacteria to support their own existence within the gut. Meanwhile, the regular development and differentiation of T cells are also efficiency in shaping microbiota and are required for maintaining intestinal microbial homeostasis.

#### Relationship between Bacteria and SIgA Secretion

SIgA is the most abundant antibody in the intestinal mucosa (65, 66). It consists of IgA dimers and a polymeric immunoglobulin receptor-derived polypeptide termed secretory component which is secreted by enterocytes to stabilize the structure of SIgA and to anchor SIgA to mucus (67). Follicles of PP and MLN are the major suppliers of SIgA. In general, most SIgA are produced in T cell-dependent way (65, 68).

When stimulated by bacterial antigen, IgA^+^ B cells in PP transfer to the intestinal stromal layer *via* lymphocyte homing to produce and secrete IgA into intestinal lumen. Then SIgA can aggregate potential and invasive pathogens to facilitate clearance of pathogens through intestinal peristalsis and mucosa cilia movement. In addition, SIgA–pathogen complex can be engulfed by M cells and recognized by DC to enhance the immune response. The complex can also combine with T cells to induce IL-4, IL-10, and TGF-β production (69). However, the mechanism of how the SIgA distinguishes commensal bacteria from harmful bacteria is still unknown and more researches are needed to explore the underlying pathway.

As discussed before, the bidirectional function does exist between bacteria and T cells. Nevertheless, T cells may mainly act as the helper of B cells to promote the IgA production (59). Some studies also confirmed the relationship between bacteria and IgA. IgA could respond to SFB and *E. coli* MG1655 with different specificities and diversification profiles (70). Moor et al. proposed another pattern of IgA-pathogen complex formation, where IgA-mediated cross-linking enchained daughter cells to form clumps eventually which could accelerate the clearance of pathogen from gut lumen and was efficient at all realistic pathogen densities (71).

Meanwhile, IgA is also a vital contributor to support the host–bacteria homeostasis. In the absence of IgA, activation-induced cytidine deaminase-deficient mice (AID−/− mice) had more Firmicutes with increased SFB (72). It was also reported that a γ-Proteobacteria-specific IgA response was in part regulated by the transition from neonatal to mature bacteria (73). Presently, an in-depth study found that IgA-mediated intestinal homeostasis and altered bacterial composition were directed by MyD88 signaling in gut T cells (74). Furthermore, confining the growth and inflammatory response of gut symbiotic flora and maintaining their diversity might be the two potential mechanisms of IgA regulating bacteria homeostasis (75).

## Regulation of Nutrition on Intestinal Bacteria and Mucosal Immunity

### Nutrients Supply to Bacteria and Modulate Their Composition

With the advancement of knowledge, very fascinating questions arise on host–microbe axis, particularly relating to nutrients that contribute to regulating bacteria composition and maintaining the homeostasis of intestinal microenvironment. A wealth of researches suggest that different diets provide the energy needs for bacteria proliferation and foster the distribution of distinct microbial communities. Besides, given many studies, deficiency and imbalance of macronutrients, mainly carbohydrate, fat, and protein, can lead causes of metabolic diseases, inducing obesity and insulin resistance (76), and can also adversely impact the intestinal bacteria. It is not to be ignored that nutrition intervention is also the alternative protocol to ameliorate immune imbalance by altering gut bacterial community nowadays.

#### Carbohydrate

Generally, the primary nutrient source for the gut bacteria is non-digestible dietary carbohydrates (NDC), which includes resistant starch (RS), non-starch polysaccharides (NSP), oligosaccharides, and unabsorbed sugars and sugar alcohols (1). Among NDC, RS and NSP are major bacterial carbon nutrients. Of course, sloughed epithelial cells and secreted mucus from intestine are also the vital fuel for energy supply for bacteria (1). The fermentation of NDC by bacteria leads to the production of short-chain fatty acid (SCFA), mainly acetate, propionate and butyrate. Meanwhile, by the intervention of NDC, the composition of saccharide-degrading bacteria is also altered. As reported, high-RS diets could induce populations of *Ruminococci* and inulin-derived prebiotics might increase the proportion of *Bifidobacteria* and *Faecalibacterium prausnitzii* (77). Meanwhile, reduced availability of NDC lowered bacterial diversity and the concentration of fiber-degrading bacteria, such as *Bacteroides ovatus, Eubacterium rectale*, and increased mucin-degrading bacteria (78). However, the inconsistent outcomes about the effects of NDC on intestinal microbial composition also have been observed according to several studies (79, 80). We suggest that the discrepant response of intestinal bacteria to NDC deprivation may depend on the complexity of bacteria composition and differences in pre-experiment individual bacteria.

A postulation in a report suggested that high diversity-fiber diets could induce high bacteria diversity (81). In addition, Chen et al. conducted a study to explore the fermentability of two fibers with different chemical structures by two fiber-utilizing bacteria, *Prevotella* and *Bacteroides*, and found that *Prevotella*-dominated bacteria instead of *Bacteroides*-dominated bacteria, could degrade quantitatively more fiber and produce more SCFA, especially propionate. After fermenting fiber substrates by fecal bacteria *in vitro*, the proportion of *Prevotella* was increased. In addition, the alteration in the *Bacteroides-*dominated enterotype group was dependent on fiber structure to a great extent, which suggested that *Bacteroides* possess higher substrate specificity than *Prevotella* (82).

However, in the large intestine, there are discrepancies on nutrient utilization among different bacteria. *Bacteroides, Prevotella, Clostridium* cluster XIV and IV are considered the carbohydrate-utilizing bacteria, in addition, the *Bacteroides* genus can also degrade mucus (1).

#### Dietary Fat

Dietary fat has been suggested to be the major contributing factor for gut bacteria modification rather than protein/sucrose ratio (83). Many studies reported that high-fat diet increased the proportion of Firmicutes and lowered the proportion of Bacteroidetes, especially *S24-7* and *Bacteroides* (84–86). This conclusion was also supported by other researches where similar outcomes were reported: greater *Enterobacteriaceae* populations in pigs fed a high-fat diet and greater *Lactobacilli, Bifidobacteria*, and *Faecalibacterium prausnitzii* in pigs fed a low-fat diet (87).

#### Protein

As to protein, excessive intake of protein always lead to higher colonic input (88). The degradation of excess proteins in colon start with hydrolysis of proteins into smaller peptides and amino acids (AA) by bacterial proteases and peptidases that are more active at neutral to alkaline pH. These residual proteins not only elevate intestinal pH but are also potentially available to the colonic microbes for further metabolism (89). The community of proteolytic bacteria is mainly altered. It has been reported that the primary bacteria related to protein metabolism in the small intestine consist of *Klebsiella* spp., *E. coli, Streptococcus* spp., *Succinivibrio dextrinosolvens, Mitsuokella* spp., and *Anaerovibrio lipolytica* (90). However, in the large intestine, the proteolytic activity of monogastric animals has been mainly attributed to the genera of *Bacteroides, Propionibacterium, Streptococcus, Fusobacterium, Clostridium*, and *Lactobacillu* (91). These dominant bacteria are not only capable to secrete various proteases and peptidases to degrade proteins, some of them could also directly metabolize AA. Furthermore, *Prevotella ruminicola, Butyrivibrio fibrisolvens, Mitsuokella multiacidas*, and *Streptococcus bovis* could secrete highly active dipeptidyl peptidase for protein digestion and absorption in the monogastric animals which community increased in the intervention of high protein level. With the participation of these proteolytic bacteria, branched-chain amino acids (BCAA), biogenic amines, and other metabolites (1, 92) from aromatic AA, such as phenylacetic acid, phenols, and indoles (93), could be derived in the process of protein fermentation.

Unlike single nutrient excesses or insufficiencies described above, malnutrition is an unsound state characterized by long-term inadequate or excessive nutrition. In fact, undernutrition is the main factor of death in children under 5 years old (94), while overnutrition is one of the chief culprits leading to obesity, diabetes, and metabolic syndrome and both have been associated with alterations in bacterial populations. *Enterobacteriaceae, Neisseriaceae* (Proteobacteria) and *Streptococcaceae* (Firmicutes) are enriched in association with undernutrition. *Bifidobacteriaceae, Coriobacteriaceae* (Actinobacteria), *Prevotellaceae* and *Bacteroidaceae* (Bacteroidetes), *Clostridiaceae, Eubacteriaceae, Lachnospiraceae, Lactobacillaceae, Ruminococcaceae*, and *Veillonellaceae* (Firmicutes) are reported to be depleted in innutrition (94, 95). Most are dominant bacteria in healthy gut. Hence, malnutrition will result in radical change of bacteria that is adverse to nutrition utilization and intestinal homeostasis to defend pathogens infection.

### Nutrients at the Interface of Host Immunity

#### Functional Amino Acids

**Figure 3:**
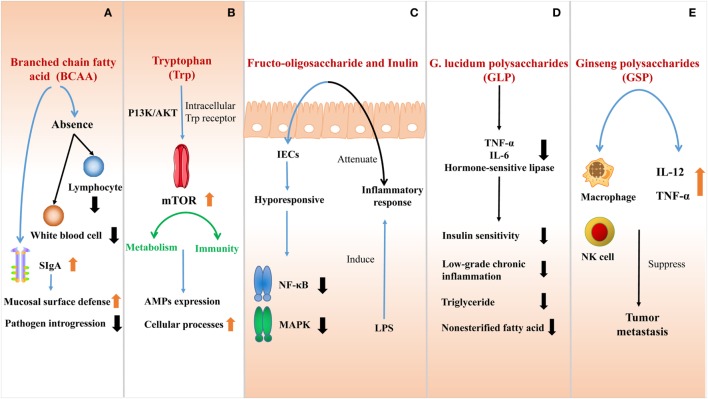
The effect of nutrients at the interface of host immunity. **(A)** The absence of branched-chain amino acids (BCAA) impairs the innate immune function due to the shortage of lymphocytes and white blood cells. BCAA can also stimulate the secretion of SIgA to improve the mucosal surface defense and inhibit pathogen introgression into the lamina propria. **(B)** Trytophan (Trp) is absorbed by intestinal epithelial cells (IECs) directly activates the mTOR pathway by intracellular Trp receptors through a PI3K/AKT independent mechanism. The active mTOR connects metabolism and immunity, promoting cellular processes, and regulating AMPs expression. **(C)** Fructo-oligosaccharide and inulin are considered as prebiotics, affecting IECs to be hyporesponsive to activate nuclear factor kappa-light-chain-enhancer of activated B cells (NF-κB) and mitogen-activated protein kinase (MAPK) induced by pathogens. Inflammatory response to lipopolysaccharide (LPS) would also be attenuated in this process. **(D)** G. lucidum polysaccharides (GLP) can effectively ameliorate the sensitivity of insulin, reducing low-grade chronic inflammation and inhibiting the outflux of plasma triglyceride and non-esterified fatty acid by suppressing the expression of tumor necrosis factor-a (TNF-α), interleukin-6 (IL-6), and hormone-sensitive lipase. **(E)** After ginseng polysaccharides (GS-P) treatment, the secretion of tumor necrosis factor-α (TNF-α) and interleukin-12 (IL-12) are increased and the macrophage and NK cell are activated. They both suppress the tumor metastasis.

Increasing evidences confirm that the intestinal immune and barrier functions are modulated by nutrients, while functional AA are prominent factors among them (Figure 3).

BCAA are EAA in mammals, which play vital roles in innate immunity as an indispensable nutrient maintaining the function of immune system. The absence of BCAA, especially isoleucine (Ile), impairs the innate immune function in cells or organisms (96), which is due to the shortage of lymphocytes and white blood cells. Concerning the gut, BCAA can also stimulate mucosal immunity and maintain intestinal integrity. Studies illustrate that the addition of BCAA limits intra-epithelial lymphocytes and decreases immunoglobulin concentration in the small intestine. BCAA stimulates intestinal SIgA secretion, which is a main immunoglobulin to improve the mucosal surface defense (97). The high amount of SlgA in the intestinal lumen is expected to be a better protection to inhibit pathogen introgression into the lamina propria (98).

Tryptophan is one of the EAA which cannot be synthesized independently by humans and animals, thus, need to ingest from food. Nowadays, several researches suggest that tryptophan seems to become a promising new target to modulate immune responses. Trytophan absorbed by IECs directly activates the mTOR pathway by intracellular tryptophan receptors through a PI3K/AKT-independent mechanism (99, 100). As we known, mTOR plays an important role in connecting metabolism and immunity. Active mTOR functions on promoting cellular processes and regulates AMPs expression (96) while suppressed mTOR reduces nutrition biosynthesis and increases autophagy (101). Increasingly large numbers of studies also show the beneficial effects of tryptophan on IBD development, which may serve as a potential candidate for treating IBD due to its benefit on intestinal barrier.

#### Polysaccharide

Many bioactive polysaccharides from various sources have been characterized, which are macromolecular carbohydrates comprised of monosaccharides serving as the most important components of organisms. As reported, polysaccharides are widely involved in antitumor, antidiabetic, antioxidant, antiviral, and immunomodulatory activities. They are also efficient of multiple physiological activities, such as cell differentiation, proliferation, and signal transduction (102–104) (Figure 3).

Fructo-oligosaccharide and inulin are considered as prebiotics, which consumption can induce immune-modulatory effects. However, this modulatory function is traditionally thought to reflect microbial interactions within the gut, which is contradictory with the recent evidence that non-digestible oligosaccharides directly regulate host in microbe-independent mechanism. In the process of directly regulating host mucosal signaling, IECs are hyporesponsive to activate NF-κB and mitogen-activated protein kinase (MAPK) induced by pathogens, when exposed to oligosaccharides. A differential kinome profile is observed when compared to those cells belonging to multiple innate immune signaling pathways. In addition, administrating non-digestible oligosaccharides orally can attenuate inflammatory response to LPS without changing intestinal microbiota. Thus, oligosaccharides can act as a kind of potent candidates to regulate host inflammation *via* directly modulating kinome instead of altering gut microbiota (105).

G. lucidum polysaccharides (GLP) can effectively ameliorate the sensitivity of insulin. After GLP treatment, insulin concentration in plasma is decreased and the systemic insulin resistance can be reversed. In this process, GLP is also potent in dampening low-grade chronic inflammation and inhibiting the outflux of plasma triglyceride and non-esterified fatty acid by suppressing the expression of TNF-α, interleukin-6 (IL-6), and hormone-sensitive lipase. Therefore, by regulating inflammatory cytokines, GLP is efficient to improve insulin sensitivity.

Researchers discovered a kind of polysaccharides from ginseng leaves (GS-P) in China recently. More importantly, tumor metastasis can be suppressed after GS-P treatment *via* activating macrophage and NK cell. Meanwhile, the secretion of TNF-α and IL-12 is also increased in peritoneal exudate macrophages (106).

In addition, β-glucans are kinds of natural polysaccharides and biologically active fibers with proven medical significance as potential probiotics. Being confirmed *in vitro*, as well as animal- and human-based clinical studies, taking β-glucans orally is of vital effect on antitumor, anti-inflammatory, anti-obesity, anti-allergic, anti-osteoporotic, and immunomodulating activities (107).

Moreover, the multi-omics approach is applied to investigate impacts of longan polysaccharide on host immune system. Results show that the level of IgA, IgG, IgM, IL-6, IFN-γ, and TGF-β is increased, which means an improvement of immunomodulatory activity.

## Nutrients Mediate the Bacteria–Immune Crosstalk through Microbiome-Modulated Metabolites

A large repertoire of metabolites of bacteria harbor in the mammalian intestine and associated mucosal immune system. They are usually small molecules with biological activity which are mainly produced from nutrition-related sources or endogenously (108).

Nutritional composition tremendously impacts the generation and scale of bacterial metabolites (108). With studies focusing on diverse substrates, it appears that protein breakdown and fermentation by microbes produce ammonia, amines, phenols, and branch chain fatty acids. In addition, multiple functional amino acids are also generated in this process and involved in immune response, such as tryptophan. SCFA are mainly derived in the metabolism of carbohydrate and fiber rich diet, and then play an important role in regulating a variety of physiological activities (109).

This variety of local metabolites affect on mucosal and systemic immune maturation and are involved in multiple immune signaling pathways with the participation of macrophages, dendritic cells (DCs), T cells, and innate lymphoid cells (ILCs) (110, 111). Moreover, many systemically absorbed metabolites may reach remote organs and modulate immune responses in sterile host regions (112, 113). The type, composition, concentration, and even the misbalance of metabolites coupled with the host sensor molecules orchestrate the immune function, no matter in steady state or during disease (114, 115).

On the basis of the intimate crosstalk existing between intestinal mucosal immune system and microbiota, metabolites generated from dietary nutrients may serve as an important bridge to connect the communication and regulation among nutrient, immune and bacteria. Furthermore, this complex network may be driven by metabolite secretion and signaling (108).

### Tryptophan Mediate Immune Response through Metabolites

**Figure 4:**
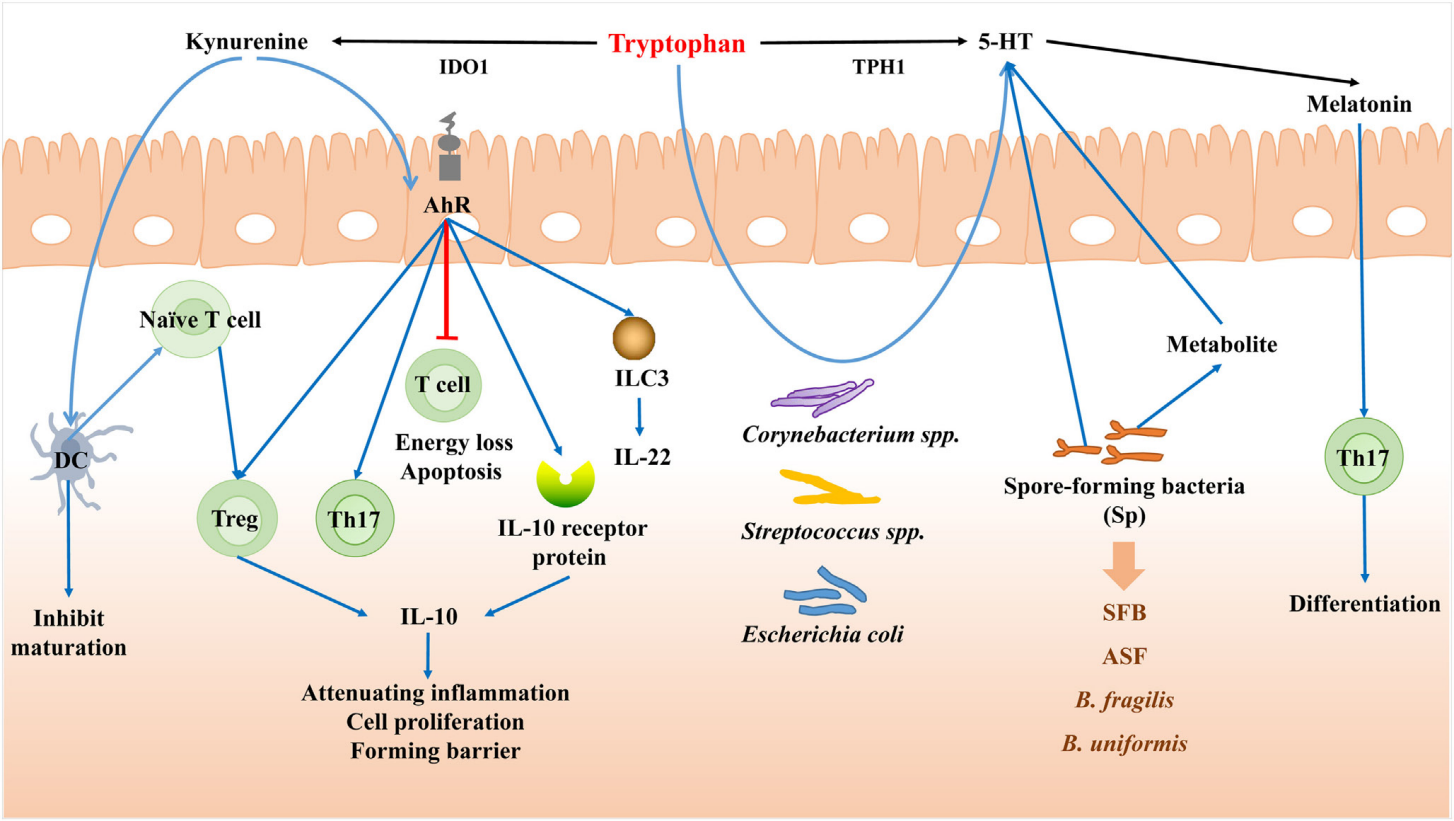
Tryptophan mediates the bacteria–immune crosstalk. When induced by proinflammatory cytokines, the majority of tryptophan is metabolized through the kynurenine (Kyn) pathway mediated by indoleamine 2,3-dioxygenase (IDO). Kyn can serve as agonists for aryl hydrocarbon receptor (AhR), and act on dendritic cell (DCs) to inhibit DCs maturation. Through AhR, Kyn can cause T cell energy loss and apoptosis, promote the proliferation of Treg and Th17 cells, and also decrease the differentiation of highly inflammatory Th17 cells and enhance the generation of IL-22 and IL-10. Tryptophan can be degraded to 5-HT by the aid of Tryptophan hydroxylase gene (TPH1). *Corynebacterium* spp., *Streptococcus* spp., and *Escherichia coli* can also synthesize 5-HT themselves from tryptophan. Spore-forming bacteria (Sp) can regulate the concentration and synthesis of 5-HT. Melatonin can be then secreted after 5-HT, which can affect the differentiation of Th17 cells in the intestine.

Tryptophan is essential for human activity and animal production. Besides the part of tryptophan that is utilized to synthesize protein, the other portion is catabolized to produce variety bioactive compounds, such as kynurenine, serotonin, melatonin, and so on, to regulate physiology functions and correspond to immunal response (Figure 4).

#### Kynurenine

When induced by proinflammatory cytokines, the majority of tryptophan is metabolized through the kynurenine (Kyn) pathway mediated by indoleamine 2,3-dioxygenase (IDO) in mammal intestine. Meanwhile a range of active metabolites known as Kyn are generated involving in immune response. Kyn itself is a compound which is almost devoid of biological activity as regarded, but can serve as the ligand for aryl hydrocarbon receptor (AhR) (116).

Aryl hydrocarbon receptor was originally identified as a dioxin detoxifying enzyme, a cytoplasmic transcription factor that was activated by many kinds of compounds (117). Kyn can serve as agonists for AhR receptor (118), and act on DCs and inhibit DCs maturation, which further control immune surveillance *via* major APC (119). Moreover, through AHR, KYN can cause T cell energy loss and apoptosis and also promote the proliferation of Treg and Th17 cells, as well as alter the response of Th1/Th2. Studies also found that coculturing with DCs and naïve T cells in the absence of AhR suppressed the later differentiation of Treg cells. However, adding Kyn into this system can redeem this effect to a certain extent and also decrease the differentiation of highly inflammatory Th17 cells and enhance the generation of IL-22 and IL-17 (120, 121). When focus on the intestinal immune system, the enhancement of IL-22 by ILC3 (122) and IL-10 receptor protein in IECs (123) is observed in the involvement of Kyn-derived AhR ligands. It has been demonstrated that IL-10 signaling in the epithelium is vital in promoting cell proliferation and forming barrier (124, 125). The deficiency of AhR or its receptor may cause spontaneous colitis (126) and destroy the intestinal homeostasis.

#### 5-Hydroxytryptamine [Serotonin (5-HT)]

Besides its character as an important neurotransmitter, the 5-HT is also a potent secretagogue and a significant regulatory factor in digestive tract with manifold biologic functions, including the mediation of intestinal secretion and motility (127–129). The majority of 5-HT is released from EC cells, which is not only able to adjust diversified physiological function in GI (127) but also expands its function in modulating immune and communicating to mucosal immune cells closely (127, 130). Immune cells of innate and adaptive system are associated with various 5-HT receptors (131), including lymphocytes, monocytes, macrophages, T cells, B cells, and dendritic cells (132).

The level of serum 5-HT can be affected by gut microbes, which was emphasized recently. In GF mice, concentrations of 5-HT are substantially reduced comparing to the conventionally colonized control group (128, 133). Indigenous spore-forming bacteria from the healthy individual enhance the level of 5-HT due to promoting its biosynthesis in colonic EC cells and then release 5-HT to mucosa and lumen (134). In addition, metabolites derived from Sp are also capable for colonic ECs to promote 5-HT biosynthesis (128). Thus, it can be demonstrated the direct regulatory effect of Sp on the concentration and synthesis of 5-HT. In addition, some specific bacterial strains, such as *B. fragilis, B. uniformis, SFB*, and *altered Schaedler flora* (*ASF*), can effectively alter the level of 5-HT in both colon and serum. *Corynebacterium* spp., *Streptococcus* spp., and *E. coli*, can synthesize 5-HT themselves from tryptophan *in vivo* (135).

Furthermore, the alteration of 5-HT mediated by microbiota regulates intestinal microenvironment. Specifically, the disturbance of enteric flora can cause the imbalance of 5-HT levels while the exertion of probiotics can significantly alleviate the symptoms of 5-HT dysbiosis (133). Thus, targeting bacteria may be serve as a prefer method to regulate peripheral 5-HT bioavailability and treat disease symptoms (128).

#### Role of Melatonin and Other Metabolites

Melatonin can be synthesized from tryptophan, which is produced from the pineal gland and the enterochromaffin cells of the intestine mucosa (136). As reported, melatonin can act as a potential candidate to treat IBD for its immunomodulatory function. Specifically, after melatonin treating, the differentiation of TH17 cells is observed to be regulated in the intestine (137). The intestinal commensal bacteria can also catabolize tryptophan to indole, indole 3-propionic acid (IPA) or indole-3-aldehyde (I3A). Recently, a study revealed that except *Clostridium sporogenes*, there were other four bacteria, *Peptostreptococcus anaerobius* CC14N and three strains of *Clostridium cadaveris* utilizing tryptophan to produce IPA, because all these bacteria encode the phenyllactate dehydratase. The study successfully modified the metabolic output by bacterial genetic engineering to regulate the intestinal homeostasis at the first time. In the context of the study, the metabolic pathways of tryptophan in *Clostridium sporogenes* were demonstrated and the generated IPA was verified to decrease the intestinal permeability. The result was consistent with a previous study, which suggested that IPA could promote the intestinal barrier function *via* PXR and TLR4 pathway (138). Apart from IPA, tryptophan can be catabolized by *lactobacilli* to I3A to prevent the colonization of *Candida albicans* and protect against mucosal inflammation through AhR recognition.

### Carbohydrates Mediate Immune Response through SCFA

SCFAs, including acetate, propionate, and butyrate, can provide energy for colonic epithelial cells and decrease luminal pH to inhibit the growth of pathogens (139). Except for aforementioned fundamental function, SCFAs can regulate the intestinal immunity in different ways. First, SCFAs can affect the intestinal distribution of immune cells. It is reported that butyrate negatively regulates the number of three ILCs in terminal ileal PPs, then results in the increment of Treg cell (140).

Second, innate and adaptive immunity can also be tuned. Because there are so many reviews that have summarized the function of SCFAs on body immune up to now (141–143), we collect the SCFA-related reports in last 2 years to present their latest progresses. Butyrate is reported to improve the expression of TLR5 to enhance the flagellin-induced immune responses through regulating the binding of Sp to TLR5 promoter (144). Propionate and butyrate can inhibit the CD8+ T cell activation to tolerate the immunity stimulation through dampering the secretion of IL-12 from DC (145). A recent study also showed that the acetate induced B cells to produce IgA *via* the retinoic acid pathway and the SCFA receptor GPR43 *in vitro* and *in vivo* (146). Similar to this study, mixed SCFAs (acetate, 70 mM; propionate, 30 mM; butyrate, 20 mM) could improve the IgA and IgG responses by regulating the gene expression for antibody production in B cells (147). However, in contrary to the results which demonstrated that butyrate mainly enhanced the acetylation of Foxp3 (148), a recent report suggested that the effect of butyrate on immune system depended on its concentration and its microenvironment. They found that lower butyrate could improve the Treg cell differentiation with the help of TNF-β while higher butyrate induced the expression of Th1-associated factor T-bet and IFN-γ, which was detrimental for intestinal mucosa homeostasis (149). At the same time, butyrate is found to increase the LL37 (an AMP) resistance of enterohemorrhagic *Escherichia coli* (EHEC) by enhancing the expression of *ompT* in EHEC and then producing more outer membrane vesicles. This phenomenon may be explained to the adaptation of EHEC to luminal environment for maintaining its survival. Hence, the role of SCFAs in intestinal homeostasis varies with their concentration and luminal microenvironment. In addition, SCFAs can protect against food allergy cooperating with vitamin A, which indicate the synergistic function of SCFAs and vitamin A on immunology regulation (150).

Generally, increment of luminal SCFAs relies on dietary fiber supplementation, and high-fiber diet produces more SCFAs. But it is unknown how much fiber is converted to SCFA. A study performed a stable isotope study to determine the systemic availability of SCFA by administrating ^13^C-labeled SCFAs into colon with colon delivery capsules. And they found 36% acetate, 9% propionate, and 2% butyrate from colon entered into circulation. This research is conducive to quantify SCFA production from ^13^C-labeled fibers by determining the systemic SCFA concentration in the future to determine the availability of colonic bacteria on fiber and then to achieve the accurate supplementation of dietary fiber in disease treatment.

## Conclusion

The available research demonstrates the interplay of bacteria–immune and nutrient-regulatory role in intestine. Additional in-depth studies are needed to uncover the underlying mechanisms of these interactions. The identification of specific nutrients involved in bacteria regulation and specific commensal bacteria involved in immunological regulation may allow the development of novel nutrient regulators used in clinical medicine as a method for the treatment of nutrient-bacteria-immune-related diseases.

## Author Contributions

The review was mainly conceived and designed by XM. Literatures were collected and analyzed by NM, JZ, and PG. The manuscript was mainly written by NM, and edited by PG, JZ, TH, SK, GZ, and XM. XM resourced the project. All the authors contributed to read and approved the final manuscript.

## Conflict of Interest Statement

The authors declare that the research was conducted in the absence of any commercial or financial relationships that could be construed as a potential conflict of interest.

## Abbreviations

AhR, aryl hydrocarbon receptor; AMP, antimicrobial peptides; AP-1, activator protein 1; APC, antigen-presenting cells; AIM2, absent in melanoma 2; BCAA, branched-chain amino acids; CLR, the C-type lectin receptors; CT, cholera toxin; DC, dendritic cells; Dvl-1, disheveled 1; ETEC, enterotoxigenic *Escherichia coli*; GALT, gut-associated lymphoid tissue; GF, germ-free; GLP, G. lucidum polysaccharides; GPR43, G protein-coupled receptor 43; IBD, inflammatory bowel disease; IDO, indoleamine 2,3-dioxygenase; IECs, intestinal epithelial cells; IFN-β, interferon-β; IL-6, interleukin-6; ILCs, innate lymphoid cells; ILF, isolated lymphoid follicles; Isg56, interferon-stimulated gene 56; IPA, indole 3-propionic acid; IRF3, interferon-regulatory factor 3; Kyn, kynurenine; Ld, *Lactobacillus delbrueckii* subsp. delbrueckii TUA4408L; LPS, lipopolysaccharide; M cells, microfold cells; MAMP, microorganism-associated molecular patterns; MAPK, mitogen-activated protein kinase; MDP, muramyl dipeptide; MHCII, major histocompatibility complex II; MLN, mesenteric lymph nodes; MYD88, myeloid differentiation primary response 88; NDC, non-digestible dietary carbohydrates; NF-κB, nuclear factor kappa-light-chain-enhancer of activated B cells; NLRC, nuclear oligomerization domain proteins subfamily C; NLRP, nucleotide-binding oligomerization domain, leucine rich repeat and pyrin domain containing; NLR, NOD-like receptors; NOD1, nucleotide-binding oligomerization domain protein 1; NSP, non-starch polysaccharides; OAS, the 2’-5’oligoadenylates synthesis; OLR, OAS-like receptors; PD-1, Inhibitory co-receptor programmed cell death-1; PEMs, peritoneal exudate macrophages; PP, peyer’s patches; PRR, pattern recognition receptors; PSA, polysaccharide A; PXR, pregnane X receptor; RIG-I, the retinoic acid inducible gene 1; RIP2, receptor-interacting protein family 2; RLRs, RIG-I-like receptors; ROS, reactive oxygen species; RS, resistant starch; SAA, serum amyloid A; SCFA, short-chain fatty acid; SED, subepithelial dome area; SFB, segmented filamentous bacteria; SIgA, secretory immunoglobulin A; TGF-β, transforming growth factor-β; TIR, toll-IL-1 receptor; TNF, tumor necrosis factor; TLR, toll-like receptors; ZPS, zwitterionic capsular polysaccharides.
